# Appropriation of the Front-of-Pack Nutrition Label Nutri-Score across the French Population: Evolution of Awareness, Support, and Purchasing Behaviors between 2018 and 2019

**DOI:** 10.3390/nu12092887

**Published:** 2020-09-22

**Authors:** Barthélemy Sarda, Chantal Julia, Anne-Juliette Serry, Pauline Ducrot

**Affiliations:** 1 Santé Publique France, French National Public Health Agency, F-94415 Saint-Maurice, France; barthelemy.sarda@santepubliquefrance.fr (B.S.); anne-juliette.serry@santepubliquefrance.fr (A.-J.S.); 2 Sorbonne Paris Nord University, Inserm U1153, Inrae U1125, Cnam, Nutritional Epidemiology ResearchTeam (EREN), Epidemiology and Statistics Research Center, University of Paris (CRESS), 93000 Bobigny, France; julia@eren.smbh.univ-paris13.fr; 3 Public Health Department, Avicenne Hospital, Assistance Publique des Hôpitaux de Paris (AP-HP), 93000 Bobigny, France

**Keywords:** front-of-pack labeling, Nutri-Score, awareness, support, purchasing behaviors, French consumers

## Abstract

Since the implementation of the Nutri-Score on a voluntary basis in 2017 in France, very few studies have evaluated how the label was recognized and used by consumers. The goal of this study was to assess the evolution of awareness, support, and perceived impact on purchasing behaviors of the Nutri-Score in France. Between April 2018 and May 2019, a total of 4006 participants were recruited across three successive waves and answered questions regarding awareness of the Nutri-Score, support of the measure, and change of behavior following the implementation of the Nutri-Score via an online survey. Descriptive analyses to assess the evolution over time were performed, as well as logistic regression models to evaluate associations between the different outcomes and individual characteristics. From April 2018 to May 2019, the awareness of the Nutri-Score increased considerably, reaching 81.5% in May 2019. Since April 2018, a steady proportion of participants—9 out of 10—showed strong support toward the measure and a similar proportion, 87.2%, declared being in favor of making the Nutri-Score mandatory. The impact on purchasing behaviors appeared promising given the limited implementation of the label, with 42.9% of the participants reporting they modified their purchasing behaviors thanks to the measure. Multivariate analyses showed that the impact on purchasing behaviors of the Nutri-Score was greater over time, on younger populations and on frequent labeling readers. Our results suggested that the labeling system was well received and used by all socioeconomic groups, including subgroups who are more likely to have a lower-quality diet.

## 1. Introduction

Noncommunicable chronic diseases, such as cardiovascular diseases, cancers, and diabetes, have become one of the major burdens to healthcare systems in industrialized countries [1,2]. In 2016, they were estimated to be responsible for 7 out of every 10 deaths in the world [3]. One of the major leading risks factors in the development of these conditions is a poor-quality diet [4]. Improving the overall quality of the diet across the population represents, therefore, a key lever in public health actions.

Different strategies have been proposed to improve the global quality of food consumed. Among them, major interest has been granted for the implementation of front-of-pack labeling (FoPL) according to the World Health Organization and the Organization for Economic Co-operation and Development (OECD) [5,6]. In Europe, therefore, there has been rising attention from public health authorities regarding FoPL [7] as it is considered an effective measure at a relatively low cost [8]. Several FoPL systems have been launched in different European countries, falling either into nutrient-specific labels, which partly reproduce the back-of-pack nutritional declaration or into summary indicators, which synthetize the nutritional information to give an overall appraisal of the nutritional quality [9]. However, there have been growing concerns that the multiplicity of formats on the EU common market could confuse consumers. In May 2020, the European Commission has engaged to propose a harmonized and mandatory FoPL by the end of 2022, thus reopening discussions regarding the food labeling policy, as defined in the 1169/2011 regulation on the provision of food information to consumers [10].

Since 2017, the Nutri-Score has been adopted by a growing number of European countries, with France, Belgium, Switzerland, Germany, and Spain having officially adopted the label, and European consumer associations showed strong support toward the measure [11,12]. This summary and interpretative FoPL classifies food products into five categories on a color-coded graded scale (ranging from “dark green”, for the “higher nutritional quality”, to “dark orange” for “lower nutritional quality”). Letters (ranging from A to E) have also been added to improve readability. The classification in each category is based on the Food Standard Agency (FSA) nutrient profiling system (NPS) [13,14] adapted for the French context, namely the FSAm-NPS. This NPS allocates points, on a 100 g or mL basis, depending on whether a nutrient or component should be encouraged or reduced.

The Nutri-Score has two specific objectives. First, it provides consumers a summarized nutritional information in a clear and understandable format, to guide them toward healthier food choices [15]. Then, as a discriminating criterion between brands, it incentivizes food manufacturers to reformulate and improve the nutritional quality of their existing and new products [16,17].

Many studies have been conducted to validate the Nutri-Score scoring system, as well as its graphical format (formerly called the 5-Color Nutrition Label or 5-CNL). The FSAm-NPS underlying the Nutri-Score has been indeed shown to be able to discriminate products across the same food category and to be consistent in regard to the French and several other European national dietary guidelines [18,19,20]. Its ability to describe food intakes has been also validated in several observational studies [21,22]. In addition, a higher score was prospectively associated with an increased risk of developing chronic diseases [23,24,25]. In both French and European contexts, the label was well perceived and enhanced consumers ability to identify healthier products [26,27,28,29,30]. Then, it has been demonstrated to improve consumers’ food choices and the nutritional quality of food baskets when purchasing [31,32,33,34,35,36]. Finally, according to a simulation study, the use of the label has been shown to reduce mortality from nutrition-related chronic disease thanks to healthier dietary intakes [37].

Since the measure was adopted on a voluntary basis, following current EU regulation, one challenge for public health authorities was to allow consumers to identify this label as the official one and to increase their awareness about this measure, while its presence on food products was inherently partial.

Therefore, this study aims at describing the evolution of the Nutri-Score’s awareness and support in the French population between April 2018 and May 2019. Moreover, another objective was to identify lifestyle and socioeconomic factors associated with higher awareness and support, as well as with a self-reported impact on food choices.

In this study, we hypothesized that the awareness of the Nutri-Score would rise thanks to an increase in the visibility of the logo, especially in subgroups that are known to be interested in nutrition. The self-reported impact on purchasing behavior was also expected to increase in conjunction with the implementation of the logo in stores, in a relatively similar way in all subgroups given that the label has been shown to be easy to understand and use.

## 2. Materials and Methods

### 2.1. Population Study

A national and representative sample of the French population was recruited through an access panel on the Internet. An e-mail was sent to the panel to invite people to participate in a study regarding food purchasing behaviors with no mention of FoPL. Three waves of questionnaire were conducted on independent samples: 1—in April 2018, before the implementation of a national information campaign about the Nutri-Score; 2—in May/June 2018 after the broadcast of the national campaign; and 3—in May 2019, one year after the first questionnaire and before the rerun of the national campaign. The second wave also had the goal of assessing the effectiveness of the national communication campaign.

The questionnaire included questions on individual characteristics: gender, age, and occupational category (retired and unemployed people were reclassified in their last position), agglomeration size, region, household composition (number of people and presence of children aged 15 or less), educational level and monthly income of the household per consumption unit (CU). According to the OECD definition, one CU was attributed for the first adult in the household, 0.5 CU for other persons aged 14 or older, and 0.3 CU for children under 14 [38]. In addition, data regarding weight status (weight, height), purchasing behavior for food products (type of food supply chain, retailers), and self-reported frequency of reading nutrition labeling were also collected. The participants were aged 15 or above.

### 2.2. Awareness, Support, and Purchasing Behavior Regarding Nutri-Score

The awareness of the logo was assessed through two questions: “have you ever heard of the Nutri-Score logo?” (Yes/No) and the recognition of the logo after presenting the visual to the participant. If a positive answer was given for either of those two, the participant was considered to know the Nutri-Score. Then, he/she was asked where he/she did see or hear about the Nutri-Score (e.g., TV, radio, food packages).

The support of the measure was assessed by asking the participants whether they were favorable to have the logo on packaging. Participants were also asked whether they would be favorable to seeing it become mandatory.

Finally, the self-reported impact of the logo on purchasing behaviors was evaluated among participants who reported knowing the Nutri-Score by asking them whether they changed, planned to change, or did not change their purchasing behavior thanks to the measure. The evaluated behaviors were the following:

- choice of a product with a higher score on the same store shelf (e.g., choosing a plain yoghurt instead of a flavored yoghurt),
- choice of a brand with a higher score for the same food product (e.g., for lasagnas, choosing a brand with a better score instead of the usual brand),
- long-term change of some food habits (e.g., reducing the consumption of sweet products and processed meat or increasing whole grain consumption),
- renouncement of buying a product if it did not present the label,
- restriction in buying food products that present a low score.

Participants were considered to have changed their purchasing behaviors if they reported that they already changed at least one of the proposed behaviors.

The full questionnaire template including the questions used in the present study is available in Supplemental Material S1.

### 2.3. Ethical Considerations

The access-panel was implemented by an agency specialized in opinion polls, BVA Group. The questionnaire was administered to eligible respondents according to BVA Group’s ethical procedures. Personal data treatment of participants was carried out in accordance with French Law no. 78-17 of the 6th of January 1978 and European Regulation no. 2016/679, known as the General Data Protection Regulation. Participation in this study was on a voluntary basis. An electronic informed consent was obtained from each participant before starting the questionnaire. Respondents had the possibility to interrupt at any time their participation. They were given small incentives for participation and were compensated in the form of points, which they could accumulate over time and convert into gifts of different types.

### 2.4. Statistical Analyses

To allow inferences on the general population, a quota method was used based on the following variables: gender, age, occupational category of the participant and the reference person in the household (upper category: head of businesses, managerial staff, intermediate profession; lower category: employees, laborers; inactive: students, homemaker), region, and agglomeration size. In addition, weighing was applied to all analyses to take into account socio-demographic differences between the panel and the French population (data from the 2015 national census of the National Institute of Statistics and Economic Studies (INSEE)).

Pearson’s chi-square tests were used to compare individual characteristics, as well as the Nutri-Score’s awareness and support of the measure across the different waves. Logistic regression models were used to assess the relationship between awareness, the wave of the questionnaire, and individual characteristics. Similar models were used for the adherence and declared impact on purchasing behavior. Individual characteristics used were gender, age (less than 25, 25–34, 35–49, 50–64, more than 65), level of diploma (with or without high-school diploma), monthly income per consumption unit (<900 €, 900 €–1500 €, >1500 €), socio-occupational category. The frequency at which participants read the nutritional information was also included to take into account the participants’ motivation toward healthy eating. Finally, awareness of the logo was also included in the model evaluating support of the measure.

The statistical significance level was set at 5%. Analyses were carried out with the SAS software (Version 9.4; SAS Institute, Inc., Cary, NC, U.S.).

## 3. Results

A total of 4006 participants were included in the study: 1005 in the first wave, 2000 in the second one, and 1001 in the last one. The sample size was doubled in the second wave because it included a post-test of the communication campaign broadcasted in May 2018. The adjusted individual characteristics of the sample are presented in Table 1. No significant differences were found according to the wave of the questionnaire.

### 3.1. Evolution in Awareness, Adherence, and Purchasing Behaviors

Table 2 displays the evolution of awareness, support, and the impact on behaviors across time. A total of 81.5% of participants answered that they had heard or recognized the logo in 2019 versus 58.2% in April 2018. The awareness of the logo showed a significant increase after the communication campaign (+17.2 percentage points between April and May 2018) and a further significant increase in 2019 (+6.1 percentage points between May 2018 and May 2019). Among the participants who knew the Nutri-Score, the most reported sources where they had seen the label were TV in May 2018 (73% vs. 57% in April 2018) and food packages in May 2019 (60% vs. 20% in May 2018).

The high support of the measure remained stable across time with 9 people out of 10 (no significant differences) declaring they were favorable to the presence of the logo on food packaging (Table 2). In addition, almost the same proportion (87%) of the population considered that the Nutri-Score should be mandatory, despite the measure being currently voluntary. Similarly, this variable did not vary across time.

The effect of the measure on change of purchasing behavior is however limited in comparison with awareness and support. In April 2018, only 26.5% of consumers declared that they adopted at least one new behavior thanks to the label. Nonetheless, a significant increase was observed over time (+16.4 percentage points between April 2018 and May 2019).

Finally, the impact of the Nutri-Score on each individual behavior in May 2019 is displayed in Table 3. A total of 42.9% of participants who knew about the Nutri-Score reported that they already changed one of the proposed behaviors thanks to the Nutri-Score. For most behaviors, between 20% and 25% of participants declared they adapted their purchasing habits according to the logo whereas only 15.1% declared having already not bought a product because it does not affix the Nutri-Score.

### 3.2. Factors Associated with the Awareness, Support of the Nutri-Score, and Purchasing Behaviors

Results of multivariate analyses are shown in Table 4. First, in accordance with previous results, time was positively associated with awareness of the Nutri-Score. Then, participants who frequently read the nutritional declaration were also more prone to being aware of the measure. In turn, the odds of knowing the logo significantly decreased when people were 50 years old or older and belonged to a lower socioeconomic group (i.e., lower income, education level, socio-occupational category). However, awareness of the logo did not vary according to gender, household income, and level of diploma.

Regarding the implementation of the Nutri-Score, participants were more likely to be in favor if they were women, aged less than 65 years old, had a household income above 1500€, had seen or heard of the Nutri-Score before, and frequently read the nutritional information.

Finally, the impact on purchasing behaviors was positively associated with time, intermediate income, and frequent reading of nutritional information. In turn, participants aged more than 35 years old and with a higher educational level were less likely to change their purchasing behaviors.

## 4. Discussion

After its implementation in France in October 2017, the present results show how the Nutri-Score has been adopted by the population between April 2018 and May 2019. Since the first wave of questionnaires, the measure received strong support from the population that appeared to remain stable over time. In turn, an increasing part of the French population declared that they knew about the Nutri-Score, reaching 81.5% in May 2019. Modification of purchasing behaviors thanks to the label affected a more limited part of the population but gradually increased over time. Overall, support to the measure, awareness of the label, and use during grocery shopping were associated with similar sociodemographic and lifestyle characteristics.

First, this study shows the rise in the Nutri-Score’s awareness between April 2018 and May 2019 and these results are consistent with the chronology of events. Indeed, between April and May 2018, a vast national communication campaign was broadcasted, leading to an increase of 17.2 percentage points in the label’s awareness. Between May 2018 and May 2019, further increase in awareness of the logo may be explained by the growing presence of the Nutri-Score in supermarkets, on online platforms, and in advertisements. In fact, according to the French Food Observatory—Oqali, the number of products affixing the logo in store shelves went from less than 800 in May 2018 to 5700 in May 2019 [39]. Moreover, popular nutrition-information smartphone applications and websites participated in the rise of visibility by using the Nutri-Score as their reference nutritional indicator, thus allowing consumers to assess the label even if it was not affixed on the food package. Furthermore, the strong support for the measure could be explained by the fact that consumers are increasingly looking for transparency [40], but also by the label format that appears quick and easy to understand. Indeed, several studies comparing different label formats have shown higher attractiveness and liking for the Nutri-Score (or the 5-CNL) [27,29,41,42], two essential characteristics to be accepted by consumers according to the conceptual framework developed by Grunert and Wills [43].

Compared with the high support of the measure and awareness of the Nutri-Score, the self-reported impact on purchasing behaviors in May 2019 appeared more limited, with 42.9% of the participants reporting having changed at least one behavior thanks to the label. A recent review and meta-analysis, including the Nutri-Score, concluded that FoPL resulted in an increased intent to buy healthier products and had the potential to impact purchasing behavior [44,45]. However, most of the studies were held in experimental conditions and, to date, very few studies were conducted in real-conditions and focused on consequences on behaviors thanks to the Nutri-Score. In France, a large-scale randomized controlled trial (RCT) performed in supermarkets before the implementation of the Nutri-Score, during 10 weeks, on four product categories in 40 supermarkets, reported that the presence of the Nutri-Score increased the purchases of higher-nutritional-quality products by 14.4% [36]. The higher impact reported in our study might be explained by the self-reported nature of the measurement, as well as the greater number of products affixing the Nutri-Score and a higher awareness about the label due to the communication campaign and its dissemination on shelves. However, given the relatively limited implementation of the label in 2019 [39], reported impact on food choices appeared promising for the future, as the Nutri-Score will appear gradually on more food products. Regarding how the Nutri-Score impacted behaviors, consumers were more likely to adapt their choices according to the label when present rather than intentionally not choosing a product without it. The real-condition trial also found no significant differences on the number of purchased products without any FoPL after the introduction of the Nutri-Score on a part of the food category [36]. These results raise the question of the voluntary nature of the measure. If no penalty is applied in the absence of the logo, the food industry will not be incentivized to add the logo, especially on products with lower scores. However, since its launch in France, the measure was adopted by many manufacturers and retailers.

Looking at the associations between the different outcomes and individual characteristics, some disparities among subgroups of the population were observed. First, the awareness of the logo and the support of the measure were more important in the younger part of the population. The higher impact on food choices reported by younger participants is particularly interesting to attenuate intergenerational differences as older people are known to be more interested in nutrition [43] and to have better-quality diets [46,47]. Modifying positively the earliest practices is particularly effective in public health as past-behaviors have the tendency to be strong predictor for future behaviors [48]. Moreover, gender was not associated with both awareness and purchasing behaviors, suggesting that the Nutri-Score may affect both genders although women are known to be more interested in healthy eating than men [46]. As expected, we also noted that participants reading nutrition facts frequently were more likely to know and use the Nutri-Score. This is in line with the literature, in which regular nutritional-information readers were more likely to accurately use the Nutri-Score or other FoPL [27,49]. Interestingly, no or few significant differences in terms of awareness, support, and purchasing behavior were found across household income, educational level, and occupational category. The Nutri-Score was better known among higher occupational categories, but no differences were observed on support and purchases. Similarly, individuals with higher income were more likely to support the measure, but this difference was not observed for behavior. The only difference on purchases was found for educational level: individuals with a higher degree were less likely to use the Nutri-Score. This might be explained by their ability to process more complex information, such as nutritional declarations, as a higher educational level is associated with greater nutrition knowledge [50,51]. Individuals with a higher educational level are also known to be more likely to have healthier dietary habits [52,53], which reduce their need for a logo to guide them toward healthier choices. These results suggest that the Nutri-Score is used by the whole population and not only by individuals already having the necessary background to make healthy food choices. This would suggest that the Nutri-Score would therefore not contribute to the further accentuation of social inequalities in health. In line with these findings, a laboratory framed field experiment and real-condition RCT observed that the Nutri-Score reduced the overall FSA score (i.e., increase the nutritional quality) of shopping carts with similar effect across the overall population, with no additional cost to the customer [36,54]. This ability to equally affect the population may be due to the easiness and quickness with which the logo can be understood without a nutritional background whereas analytical systems, such as Multiple Traffic Lights or Reference Intake, require more processing and may create decisional conflicts, which translates to a smaller impact, particularly in the most vulnerable subgroups [26,27,28,29].

To our knowledge, the present study is the first to report data regarding the awareness of the Nutri-Score since its implementation in France in 2017. Additionally, we performed three repeated measures over time, which allows to relate evolutions in regard to the implementation of the label. These data may be useful to better understand the appropriation of the label by the French population, in particular given the voluntary nature of the measure. However, some limitations should be acknowledged. In order to ensure good sample representativeness, the quota method was applied. This easy-to-implement method of adjustment tends to under-represent the most nutritionally vulnerable people in the population. A study showed significant differences between quota and randomized sampling, by exclusion of those more fragile populations [55]. Moreover, online polling was chosen to administer the questionnaire to participants. Although surveying on the Internet has undeniable advantages such as speed, cost, or flexibility, it may create a selection bias against people who do not have Internet access and the illiterate population [56]. Literature shows that the prevalence of women, older, and highly educated people is higher in Internet panels in comparison to that in paper-based surveys [57]. Different biases may have reduced the prevalence of vulnerable populations in the present study and led to an over-estimation of the indicators. Finally, the impact of the label on purchasing behaviors was self-reported. Social desirability bias may have led to a greater proportion of reported behavior improvement. Nonetheless, our results are useful to highlight a positive evolution of the awareness and use of the label.

## 5. Conclusions

The present study reports, for the first time, results to assess the appropriation of the Nutri-Score in the French population, allowing an overview on how the label has been received by the population two years after its launch. The logo, strongly supported by consumers, appeared to become more and more visible and thus started to influence the way people chose food products. Our results suggested that the measure similarly impacted the different socioeconomic groups, including subgroups who are more likely to have a lower-quality diet such as individuals with a lower income or a lower educational level. In addition, the Nutri-Score, by acting as a discriminating criterion between products is an incentive for manufacturers to reformulate their existing and new products, which may lead to a global improvement of nutritional quality in the food offer. In this way, every consumer may also benefit from the measure, even those who do not take nutritional quality into account. Further studies focusing on the food on offer and sales evolution are needed to provide insights into how the Nutri-Score may have affected the quality of diet, particularly in nutritionally at-risk subgroups. Finally, from a public health perspective, in order to further promote the use of the Nutri-Score by the population, implementing actions toward teenagers may be interesting, as they benefit from a certain autonomy in their food choices and have a prescribing effect on food purchases.

## Figures and Tables

**Table 1:** Individual characteristics of participants across the three successive questionnaires (weighted data).

Variable	April 2018 (*n* = 1005)	May 2018 (*n* = 2000)	May 2019 (*n* = 1001)	*p*-Value
Gender				1.0
Men	47.8%	47.8%	47.8%	
Women	52.2%	52.2%	52.2%	
Age				1.0
Less than 25	14.4%	14.4%	14.3%	
25–34	15.1%	15.1%	15.0%	
35–49	24.8%	24.8%	24.5%	
50–64	24.0%	24.0%	24.0%	
More than 65	21.7%	21.7%	22.3%	
Monthly income per CU				0.29
Less than 899 €	17.2%	16.9%	17.4%	
900 € to 1499 €	18.4%	19.5%	19.1%	
More than 1500 €	47.5%	46.5%	49.9%	
No answer	16.9%	17.1%	13.7%	
Educational level				0.51
No high-school diploma	29.1%	28.6%	30.7%	
High-school diploma or more	70.9%	71.4%	69.4%	
Occupational category				0.99
Upper category	46.6%	47.6%	48.0%	
Lower category	37.9%	36.9%	36.4%	
Inactive	15.5%	15.5%	15.6%	

CU, consumption unit. One CU is attributed for the first adult of the household, 0.5 CU for other persons aged 14 or older, and 0.3 CU for children under 14 years old. Differences for each variable were assessed with Pearson’s chi-square test.

**Table 2:** Evolution of awareness, support, and purchasing behaviors across the three successive questionnaires.

Variable	April 2018 (*n* = 1005)	May 2018 (*n* = 2000)	May 2019 (*n* = 1001)	
	Mean	Mean	Mean	*p*-Value
**Awareness**				
Heard in the past	41.5% ^a^	62.5% ^b^	69.2% ^c^	<0.001
Visual recognition	44.6% ^a^	64.2% ^b^	72.6% ^c^	<0.001
Total Awareness	58.2%^a^	75.4% ^b^	81.5% ^c^	<0.001
**Support**				
In favor of the Nutri-Score	90.4%	91.0%	90.4%	0.75
In favor of making the Nutri-Score mandatory	87.1%	86.6%	87.2%	0.41
Impact on behaviors *				
Modified at least one behavior thanks to the Nutri-Score	26.5% ^a^	29.0% ^a^	42.9% ^b^	<0.001

Differences for each variable were assessed with Pearson’s chi-square test. Letters ^a, b, c^ indicate significant differences between different waves. * Question only asked to participants who reported to be aware of the Nutri-Score.

**Table 3:** Percentages of people declaring a change in purchasing behavior in May 2019.

Behavior	Change in Behavior in May 2019 (*n* = 808 *)
Choosing a product with a better score on the same shelf (e.g., choosing a plain yogurt instead of a flavored one)	23.6%
Choosing a brand with a better score for the same food product (e.g., for lasagnas, choosing a brand with a better score instead of the usual brand)	22.9%
Long-term change of some food habits toward healthier habits (e.g., reducing consumption of sweet products and processed meat or increasing whole grain consumption)	25.0%
Renouncement of buying a product if there was no logo	15.1%
Restriction in buying food products that present a low score	23.0%

* Questions only asked to participants who reported familiarity with the Nutri-Score.

**Table 4:** Associations between awareness, support, or change in purchasing behaviors with individual characteristics and time using logistic regression models.

Variable	Awareness (*n* = 4006)		Support (*n* = 4006)		Purchasing Behaviors (*n* = 2909 *)	
	OR (95%CI)	*p*-Value	OR (95%CI)	*p*-Value	OR (95%CI)	*p*-Value
**Time**		<0.001		0.30		<0.001
April 2018	1		1		1	
May 2018	2.27 (1.93–2.68)		0.91 (0.69–1.19)		1.19 (0.95–1.49)	
May 2019	3.24 (2.64–3.99)		0.78 (0.57–1.07)		2.25 (1.77–2.86)	
**Gender**		0.41		<0.001		0.25
Men	1		1		1	
Women	0.94 (0.80–1.09)		1.50 (1.19–1.90)		1.10 (0.93–1.32)	
**Age**		<0.001		0.001		<0.001
Less than 25	1		1		1	
25–34	0.95(0.70–1.30)		0.84 (0.52–1.35)		0.81 (0.59–1.11)	
35–49	0.90 (0.68–1.21)		0.68 (0.44–1.05)		0.51 (0.38–0.69)	
50–64	0.73 (0.54–0.98)		0.90 (0.56–1.42)		0.56 (0.41–0.76)	
More than 65	0.53 (0.39–0.72)		0.47 (0.29–0.76)		0.48 (0.34–0.67)	
**Monthly income per CU**		0.95		<0.001		0.01
Less than 899 €	1		1		1	
900 € to 1499 €	0.97 (0.76–1.24)		1.35 (0.97–1.89)		1.43 (1.09–1.87)	
More than 1500 €	0.99 (0.79–1.25)		2.44 (1.76–3.40)		1.18 (0.91–1.51)	
**Educational level**		0.08		0.52		0.003
No high-school diploma	1		1		1	
High-school diploma or more	1.17 (0.98–1.39)		0.92 (0.71–1.19)		0.74 (0.60–0.90)	
**Socio-occupational category**		0.03		0.40		0.17
Upper category	1		1		1	
Lower category	0.80 (0.67–0.96)		0.84 (0.64–1.11)		0.85 (0.70–1.05)	
Inactive	1.02 (0.76–1.35)		0.78 (0.51–1.20)		1.06 (0.79–1.43)	
**Frequency of reading nutrition information**		<0.001		<0.001		<0.001
Rarely	1		1		1	
Frequently	2.04 (1.76–2.36)		2.22 (1.77–2.77)		3.76 (3.09–4.56)	
**Awareness**				<0.001		
Is not aware of the Nutri-Score	NA		1		NA	
Is aware of the Nutri-Score	NA		2.24 (1.78–2.82)		NA	

OR, odds ratio; CI, confidence interval; CU, consumption unit. One CU is attributed for the first adult of the household, 0.5 CU for other persons aged 14 or older, and 0.3 CU for children under 14 years old. NA: not applicable: awareness was not included in these models. * Questions only asked to participants who reported the Nutri-Score.

## Supplementary Materials

The following are available online at https://www.mdpi.com/2072-6643/12/9/2887/s1, Supplemental Material S1: Questionnaire template and questions used in the study.
