# Supplementary material for: Appropriation of the Front-of-Pack Nutrition Label Nutri-Score across the French Population: Evolution of Awareness, Support, and Purchasing Behaviors between 2018 and 2019
Source: Nutrients. 2020 Sep 22;12(9):2887. doi: 10.3390/nu12092887 (PMC7551644; doi:10.3390/nu12092887)
Supplement: Supplementary file 1 [file nutrients-12-02887-s001.pdf]

Questionnaire Template and Questions Used in the Study.

1. Personal information (9 questions)
2. Food shopping behaviors (6 questions)
3. Awareness of Nutri-Score (4 questions)
4. Understanding and perception of the logo (3 questions)
5. Impact on purchasing behaviors (4 questions)
6. Support of the measure (5 questions)
7. Personal information (2) (4 questions)

Elements related to programming in green :
- Mandatory answer to each question
- 1 answer per question unless specifically indicated
- No possibility of going back to already answered questions

Questions used in the study:

Personal information

To all

Z1. Are you a :

1. A man
2. A woman

Z2. What is your year of birth?

Terminals: 1900-2003

/__/__/__/__/

Recoded in 5 age groups
1. 15-24 years old
2. 25-34 years
3. 35-49 years old
4. 50-64 years old
5. Age 65 and over

Z0a. Please indicate the postal code of your municipality

/__/__/__/__/__/

If postal code does not exist, display error message blocking
If postal code exists

Z0b. In which town do you live?

List of towns according to postal code

Recoding:
One of the following regions was assigned according to Z0a.
1. *Région parisienne*
2. *Bassin Parisien Est*
3. *Bassin Parisien Ouest*
4. *Nord*
5. *Est*
6. *Ouest*
7. *Sud Ouest*
8. *Sud Est*
9. *Méditerranée*

One of the following agglomeration sizes was assigned according to Z0a.
1. *Rural*
2. *Less than 20 000 inhabitants*
3. *20 000 à 99 000 inhabitants*
4. *100 000 or more inhabitants*
5. *Paris agglomeration*

To all
Z3. What is your main activity at the moment?
1. Private sector employee
2. Employee of a public or national enterprise
3. Public sector employee
4. Self-employed
5. Looking for a first job
6. Looking for a job (you've already worked)
7. Retired
8. At home
9. Pupil or student
10. In another situation

Z3 and Z3b filters :
if Z3=1or2or3 then do not propose the modalities i1and2and3and4and12and13and14 in Z3b
if Z3=4 then do not propose the modalities i5and6and8and9and10and11and12and13and14 in Z3b
if Z3=6 then do not propose the modalities i12and13and14 in Z3b

if Z3=5or7or8or9or10 then do not ask Z3b and recode automatically :
if Z3=9 then Z3b=13
if Z3=7 then Z3b=12
if Z3=5or8or10 then Z3b=14

if Z3=1or2or3or4

Z3b. What is your profession, your socio-professional category?

If Z3=6

Z3b. Before you were unemployed, what was your last occupation, your last socio-professional category?

1. Farmer operating
2. Artisan small trader
3. Head of a company with more than 10 employees
4. Liberal profession (EXCEPT paramedical)
5. Professor / scientific profession
6. Executive and other senior intellectual profession
7. Foreman, supervisor, paramedical profession, technician
8. Teacher
9. Employee
10. Service Staff
11. Worker / Agricultural worker
12. Retired
13. Pupil / Student
14. Other inactive

Recoding:
If Z3b=1or2or3 then recodZ3=1
If Z3b=4or5or6 then recodZ3=2
If Z3b=7or8 then recodZ3=3
If Z3b=9or10 then recodZ3=4
If Z3b=11 then recodZ3=5
If Z3b=12 then recodZ3=6
If Z3b=13 then recodZ3=7
If Z3b=14 then recodZ3=8

recodZ3: PROFESSION INTERVIEWED RECLASSIFIED IN 8 GROUPS

1. Self-employed entrepreneurs
2. Executives and Senior Professionals
3. Intermediate occupations
4. Employees
5. Workers
6. Retired
7. Pupils / Students
8. Other miscellaneous inactive

If retired (Z3=7)

Z3c. Before you retired, what was your last profession, your last socio-professional category?

1. Operating farmer
2. Artisan small trader
3. Head of a company with more than 10 employees
4. Liberal profession (EXCEPT paramedical)
5. Professor / scientific profession
6. Executive and other senior intellectual profession
7. Foreman, supervisor, paramedical profession, technician
8. Teacher
9. Employee
10. Service Staff
11. Worker / Agricultural worker

To all

Z4. What is your role in the household?

1. you live alone
2. a member of the couple
3. the only parent in the household
4. a child of the family
5. a friend or relative staying with the family
6. other (roommates, household staff...)

if Z1=2 and Z4=2 then Z4b=1
if Z4=4 then Z4b=2
if Z4=5or6 then Z4b=4

Z4b Recoding head of family

1. your husband
2. your father (or your mother if your father does not live at home)
3. your parent
4. the main occupant of the household (male if a couple)

If Z4=1or3 or (Z1=1 and Z4=2) then do not set Z8 and Z8b and Z8c and recode automatically:
Z8=Z3
Z8b=Z3b
Z8c=Z3c

If Z4b=1or2or3or4

Z8. What is the main activity of [Z4b] at the moment?

1. Private sector employee
2. Employee of a public or national enterprise
3. Public sector employee
4. Self-employed
5. Looking for a first job
6. Looking for a job (has already worked)
7. Retired
8. At home
9. Pupil or student
10. In another situation

FILTERS Z8 and Z8b If Z4b=1or2or3or4 :
if Z8=1or2or3 then do not propose the modalities i1and2and3and4and12and13and14 in Z8b
if Z8=4 then do not propose the modalities i5and6and8and9and10and11and12and13and14 in Z8b
if Z8=6 then do not propose the modalities i12and13and14 in Z8b

if Z8=5or7or8or9or10 then do not ask Z8b and recode automatically :
if Z8=9 then Z8b=13
if Z8=7 then Z8b=12
if Z8=5or8or10 then Z8b=14

if Z8=1or2or3or4

Z8b. What is the occupation, the socio-professional category of [Z4b]?

If Z8=6

Z8b. Before becoming unemployed, what was the last occupation, the last socio-professional category in [Z4b]?

1. Farmer operating
2. Artisan small trader
3. Head of a company with more than 10 employees
4. Liberal profession (EXCEPT paramedical)
5. Professor / scientific profession
6. Executive and other senior intellectual profession
7. Foreman, supervisor, paramedical profession, technician
8. Teacher
9. Employee
10. Service Staff
11. Worker / Agricultural worker
12. Retired
13. Pupil / Student

14. Other inactive

If Z8b=1or2or3 then recodZ8=1
If Z8b=4or5or6 then recodZ8=2
If Z8b=7or8 then recodZ8=3

If Z8b=9or10 then recodZ8=4
If Z8b=11 then recodZ8=5
If Z8b=12 then recodZ8=6
If Z8b=13 then recodZ8=7
If Z8b=14 then recodZ8=8

RecodZ8: FAMILY HEAD PROFESSION RECLASSIFIED IN 8 GROUPS
1. Self-employed entrepreneurs
2. Executives and Senior Professionals
3. Intermediate occupations
4. Employees
5. Workers
6. Retired
7. Pupils / Students
8. Other miscellaneous inactive

If Z4b=1or2or3or4 AND Z8=7
Z8c. Before retirement, what was the last occupation, the last socio-professional category in [Z4b]?
1. Farmer operating
2. Artisan small trader
3. Head of a company with more than 10 employees
4. Liberal profession (EXCEPT paramedical)
5. Professor / scientific profession
6. Executive and other senior intellectual profession
7. Foreman, supervisor, paramedical profession, technician
8. Teacher
9. Employee
10. Service Staff
11. Worker / Agricultural worker

If Z4=1 then do not ask Z6 and automatically recode Z6=1.
If Z4>1
Z6. How many people make up your household, including yourself?
1. 1 person
2. 2 persons
3. 3 persons

4. 4 persons
5. 5 persons
6. 6 persons
7. 7 persons
8. 8 persons
9. 9 or more persons

Filter for Z7 :
if Z4=1 or Z6=1 then do not ask Z7 and automatically recode Z7=1
if Z6=2 then do not ask the modalities i3 to i10
if Z6=3 then do not ask the modalities i4 to i10
if Z6=4 then do not ask the modalities i5 to i10
if Z6=5 then do not ask the modalities i6 to i10
if Z6=6 then do not ask the modalities i7 to i10
if Z6=7 then do not ask the modalities i8 to i10
if Z6=8 then do not ask the modalities i9 to i10

Z7. How many children UNDER the age of 15 currently live in the home?

1. None
2. 1 child
3. 2 children
4. 3 children
5. 4 children
6. 5 children
7. 6 children
8. 7 children
9. 8 children
10. 9 children and more

Awareness of Nutri-Score

To all

Q7. Have you ever heard of the Nutri-Score logo, if only in name?

1. Yes, and I can see what it means
2. Yes, but I don't quite see what it is about
3. No

Q8. Here is the Nutri-Score logo. Have you ever seen this logo?

Logo display (possibility to scroll through the 5 logos with different letters)

1. Yes
2. No

Has already heard of or seen the logo (Q7 = 1 or 2 OR Q8 = 1)

Q9. Where did you hear or see the Nutri-Score logo?

Multiple answers possible

1. On television
2. In the newspapers, the press
3. On a poster
4. On the radio
5. On a leaflet, flyer, information document
6. On the packaging of a product while shopping in a grocery store
7. On the packaging of a product while shopping on the Internet on a drive or for a home delivery order
8. On the packaging of a product in another situation (e.g. in a vending machine, at a friend's house)
9. On another website
10. On an application
11. By a health professional (doctor, pharmacist, dietician, etc.)
12. By people you know
13. By other means (specify)

Impact on purchasing behaviors

To all

Currently, the Nutri-Score logo is only indicated on certain food products.

Has already heard of or seen the logo (Q7 = 1 or 2 OR Q8 = 1)

Q15. Have you personally ever purchased a food product with the Nutri-Score logo on it?

Logo display

1. Yes
2. No
3. I did not see the logo during my purchases
4. I don't know anymore

Purchased a product with the Nutri-Score logo (Q15 = 1)

Q16. You have already purchased a food product with the Nutri-Score logo on it. Did the presence of this logo on the packaging motivate the purchase of this product?

If you have already purchased several products with this logo, please answer in regard to the last product with this logo that you purchased

1. Yes, absolutely
2. Yes, rather
3. No, rather not

Has already heard of or seen the logo (Q7 = 1 or 2 OR Q8 = 1)

Q17. Can the Nutri-Score logo be used on your grocery shopping trips?

Random item rotation - make items appear one after the other

a) Make you choose a product with a better score rather than another with a worse score within the same shelf: for example, choose a plain yogurt rather than a flavoured yogurt, choose a vegetarian pizza rather than a 4 cheese pizza?
b) Make you buy another brand for the same food product: for example, for lasagna, buy a brand X with a better score rather than a brand Y with a lower score that you are used to buying?
c) Make you change your long-term eating habits: for example, consume less sweet products or cold cuts, eat more wholemeal bread...?
d) Make you renounce to buy a product if it does not affix the logo?
e) Make you limit the purchase of food products with lower scores?

1. Yes and this has already been the case
2. Yes, in the future
3. No

Support of the measure

To all

The Nutri-Score is a logo that informs consumers about the nutritional quality of a food product. The product is positioned on a 5-level scale with a color and a letter from A to E.

Q18. Would you say that this logo is useful to assess the nutritional quality of a food product?

1. Very useful
2. Rather useful
3. Rather not

4. Not at all useful

Q19. Are you in favor or not of having this logo on food packaging?

1. Strongly support
2. Rather favorable
3. Somewhat unfavorable
4. Not at all favorable

Q21. Currently each brand has the choice to put this logo on their products or not. Are you in favor or not of making this logo mandatory on all food packaging?

1. Strongly support
2. Rather favorable
3. Somewhat unfavorable
4. Not at all favorable

Q22. Do you ever read on the packaging of the food products you buy, the list of ingredients or the table showing the nutritional composition of the product?

1. Always
2. Often
3. Sometimes
4. Never

Personal information (2)

To all

Q23. What is your height?

Specify your height in centimeters (cm)
Please answer approximately if you don't know
Example: 1m50=150 centimeters
Terminals: 100-250
/__/__/__/ cm

Q24. What is your weight?

Specify your weight in kilos (kg)
If you are pregnant, please give your weight before your pregnancy.
Please answer approximately if you do not know
Terminals: 30-200
/__/__/__/ kg

Z11. What is the highest degree you have completed?

1. No Degree / Certificate of Elementary Education

2. College Certificate
3. CAP / BEP
4. BAC
5. BAC +2 or higher

Z12. Finally, could you indicate your household's net monthly income before taxes, i.e. by counting all of your household's resources: family allowances and professional income of all household members including overtime, bonuses and 13th month, as well as other financial resources of all household members: retirement pension, income from movable and immovable property.

Which of the following brackets does your household's net monthly income before taxes fall into?

1. Less than 499 Euros
2. From 500 to 749 Euros
3. From 750 to 999 Euros
4. From 1,000 to 1,499 Euros
5. From 1 500 to 1 999 Euros
6. From 2,000 to 2,499 Euros
7. From 2 500 to 2 999 Euros
8. From 3,000 to 3,499 Euros
9. From 3 500 to 4 499 Euros
10. From 4,500 to 5,999 Euros
11. From 6,000 to 7,499 Euros
12. Over 7,500 Euros
13. You don't know or don't want to say it
